# Irregular sleep and cardiometabolic risk: Clinical evidence and mechanisms

**DOI:** 10.3389/fcvm.2023.1059257

**Published:** 2023-02-17

**Authors:** Chengjie Zhang, Gang Qin

**Affiliations:** ^1^First School of Clinical Medicine, Shanxi Medical University, Taiyuan, China; ^2^Department of Cardiology, First Hospital of Shanxi Medical University, Taiyuan, China

**Keywords:** sleep regularity, sleep health, cardiometabolic diseases, circadian dysregulation, behavioral factor

## Abstract

Sleep regularity is an essential part of the multidimensional sleep health framework. The phenomenon of irregular sleep patterns is widespread in contemporary lifestyles. This review synthesizes clinical evidence to summarize the measures of sleep regularity and discusses the role of different sleep regularity indicators in developing cardiometabolic diseases (coronary heart disease, hypertension, obesity, and diabetes). Existing literature has proposed several measurements to assess sleep regularity, mainly including the standard deviation (SD) of sleep duration and timing, sleep regularity index (SRI), interdaily stability (IS), and social jetlag (SJL). Evidence on associations between sleep variability and cardiometabolic diseases varies depending on the measure used to characterize variability in sleep. Current studies have identified a robust association between SRI and cardiometabolic diseases. In comparison, the association between other metrics of sleep regularity and cardiometabolic diseases was mixed. Meanwhile, the associations of sleep variability with cardiometabolic diseases differ across the population. SD of sleep characteristics or IS may be more consistently associated with HbA1c in patients with diabetes compared with the general population. The association between SJL and hypertension for patients with diabetes was more accordant than in the general population. Interestingly, the age-stratified association between SJL and metabolic factors was observed in the present studies. Furthermore, the relevant literature was reviewed to generalize the potential mechanisms through which irregular sleep increases cardiometabolic risk, including circadian dysfunction, inflammation, autonomic dysfunction, hypothalamic–pituitary–adrenal (HPA) axis disorder, and gut dysbiosis. Health-related practitioners should give more attention to the role of sleep regularity on human cardiometabolic in the future.

## 1. Introduction

Cardiometabolic disease is the leading cause of morbidity or mortality worldwide. Sleep, a basic life activity of the human body, is necessary for the proper function of the cardiovascular system and metabolic regulation (1, 2), and is associated with the development of cardiometabolic diseases. There is extensive evidence showing a U-shaped association between sleep duration and cardiometabolic risk in populations (3). In addition, large-scale population-based surveys suggest that delayed sleep onset timing also increases the risk of cardiometabolic disease (4). However, the above indicators are usually described as averages over multiple days in studies, concealing differences in sleep duration or time points at different periods. Even under normal conditions, the body’s daily sleep–wake schedule fluctuates. The fluctuations in the sleep–wake schedule will be greater when we are troubled by sleep disorders, physical illnesses or life events. Sleep regularity reflects the degree of variation in the daily sleep situation of the human body. Studies have shown the cross-sectional association between irregular sleep patterns and various physiological functions such as circadian rhythms, endocrine, and metabolism (5–7). Furthermore, several studies indicate that irregular sleep pattern was more strongly associated with cardiovascular disease than short sleep duration (8, 9).

In modern life, irregular sleep patterns prevail among the population. Shift workers show dramatic changes in sleep patterns, and studies have found that they were at significantly increased risk of cardiovascular disease, obesity, and hypertension (10–12). Similarly, there is clinical evidence that chronic irregular sleep patterns increase the risk of cardiometabolic disease among non-shift workers (7). Therefore, a comprehensive understanding of the link between sleep regularity and cardiometabolic is critical to advancing public health.

### 1.1. Measurement of sleep regularity

Sleep regularity is also known as sleep consistency, sleep variability, or intraindividual difference in sleep. Existing literature has proposed several relevant indicators to assess sleep regularity (Table 1), and there are differences in the measurement methods of the same indicator (which can be roughly divided into instrumental measurement and self-report). The heterogeneity of evaluation metrics and measurement methods made it difficult to synthesize relevant findings. A study used machine-simulated multiple sleep–wake patterns to evaluate the value of different sleep regularity indicators. The results showed that each indicator reflected different aspects of sleep regularity (13). Social jetlag (SJL) (14) mainly measures sleep regularity throughout the week and is insensitive to day-to-day sleep variability. Composite phase deviation (CPD) (15) and sleep regularity index (SRI) (16) are called consecutive metrics and describe the variability between consecutive days. Interdaily stability (IS) (17) and intra-individual standard deviation (SD) are termed overall metrics that reflect the variability of the overall sleep situation during the monitored period. Sleep regularity questionnaire score (SRQ) is a subjective sleep regularity evaluation index correlated with patients’ emotional health, such as anxiety and depression (18). Among the multiple indicators of sleep regularity, it is unclear which one has the strongest association with cardiometabolic disease.

**Table 1:** Definition of different assessment related to sleep regularity.

Sleep regularity measure	Definition	Measurement tool	Interpretation
Standard deviation(SD)	Standard deviation of sleep duration or sleep timing within days	Actigraphy or sleep diaries or PSG	Lower numbers indicate higher regularity
Sleep Regularity Index (SRI)	Percentage probability of being in the same state (sleep or wake) at any two points 24 h apart (repeat in 30 s)	Actigraphy or sleep diaries or PSG	Higher numbers indicate higher regularity
Interdaily stability (IS)	Rest–activity rhythms over multiple days, calculated as the ratio of the variance within the same time interval each day and the overall variance	Actigraphy or PSG	Higher numbers indicate higher regularity
Composite Phase Deviation (CPD)	Combining ΔChronotype (the difference between sleep midpoint on 1 day and Chronotype)and ΔDay-to-Day (the difference between sleep midpoint on 1 day and pervious day)	Actigraphy or sleep diaries or PSG	Lower numbers indicate higher regularity
Sleep Regularity Questionnaire Score (SRQ)	A short subjective measure of sleep regularity retrospectively in the form of a questionnaire	Questionnaire	Higher scores indicate higher regularity
Social jetlag (SJL)	The difference between sleep midpoint on freedays and non-free/workdays	Actigraphy or sleep diaries or PSG or questionnaire	Lower numbers indicate higher regularity

## 2. Sleep regularity and cardiometabolic disease

In addition to short sleep duration, sleep regularity is increasingly recognized as closely related to cardiometabolic health. Cardiometabolic diseases mainly include coronary heart disease, hypertension, obesity, and diabetes. These diseases are interconnected and affect human health together. Growing clinical evidence demonstrates that irregular sleep may be a risk factor for cardiometabolic disease.

### 2.1. Hypertension

The link between sleep regularity and hypertension has been studied in clinical settings (Supplementary Table S1). Standard deviation (SD) is most commonly used to assess sleep regularity. A population-based study of 2,598 middle-aged Swiss did not find a significant association between SD of nighttime sleep duration measured by actigraphy and the prevalence of hypertension (19). Similarly, neither cross-sectional nor prospective analysis of data from the MESA Sleep Ancillary Study found an influential association of SD of sleep characteristics (sleep duration or onset timing) with hypertension (20). In contrast to the above studies, sleep regularity quantified by SD of actigraphy-derived sleep midpoint was related to hypertension in a cross-sectional survey of 700 participants from MIDUS cohorts (21).

The Sleep Regularity Index (SRI) is a newly emerging measure of sleep regularity. In the same sample from the MESA Sleep Ancillary Study, an analysis of the association between sleep regularity as measured by SRI and hypertension yielded that lower SRI was associated with a higher prevalence of hypertension (16).

Clinical studies also considered the relationship of hypertension with interdaily stability (IS), another measure of sleep regularity. By analyzing data from 156 adults aged 18 to 64, we found that for every 10% decrease in IS value, there was an absolute 3.0% increase in the prevalence of hypertension (22). The Rush Memory and Aging Project included 1,137 older adults and found that higher IS values were associated with a lower prevalence of hypertension (17).

Social jetlag (SJL) is an important complementary measure of sleep regularity. In a retrospective and longitudinal study of 625 patients with non-communicable chronic diseases, generalized estimating equations analysis suggested an isolated effect of SJL on diastolic BP (23). In 962 adults with pre-diabetes/untreated Type 2 diabetes, SJL was associated higher blood pressure (24). However, a cross-sectional study including 147 participants did not find a significant association between SJL assessed by the Munich Chronotype Questionnaire and hypertension (25). In addition, a prospective study of 430 healthy young adults also found no correlation between actigraphy-measured SJL and blood pressure (26). Two studies in children have yielded similar negative results (27, 28).

Collectively, association between sleep regularity and hypertension varies significantly across types of indicators. A limited number of studies showed that only SRI and IS were consistently associated with hypertension or higher blood pressure, not SJL or SD. Of note, the association between SJL and hypertension for patients with diabetes was more accordant than the general population. Differences in study results are not only related to the heterogeneity of study designs but may also be attributed to the characteristics of different indicators. Compared with the remaining indicators, the SRI and IS indicators combined all the sleep–wake information during the recording period. Therefore, SRI and IS may be more sensitive in finding the association between sleep regularity and hypertension.

### 2.2. Diabetes

Diabetes is a strong predictor of cardiovascular disease and a severe threat to human health. Previous studies have shown a correlation between sleep duration and quality and the incidence of diabetes (29). Sleep regularity is an important indicator independent of other dimensions of sleep health. Evidence is mounting that irregular sleep is involved in the development of diabetes (Supplementary Table S2). A cross-sectional analysis of 1986 elders with metabolic syndrome indicated that the standard deviation (SD) of sleep duration was positively associated with the prevalence of type 2 diabetes (30). In contrast, no significant association was observed between SD of total sleep duration and diabetes in a cross-sectional study of 771 adults or a population-based cohort of 2,598 middle-aged subjects (19, 31). Fasting blood glucose and glycated hemoglobinA1c (HbA1c) are commonly used to evaluate glucose metabolism. In the middle-aged and older adults from the MESA cohort study, a cross-sectional analysis showed that the detection rate of high fasting blood glucose increased by 20 and 30%, respectively, for each 1-h increase in SD of sleep duration and sleep onset time (20). Studies in young individuals also showed that a higher SD of sleep duration was associated with increased fasting and postprandial blood glucose (32). However, a cross-sectional study including 1986 elders found null association between SD of sleep duration and fasting plasma glucose and HbA1c (30). Insulin resistance is one of the most critical pathogenesis mechanisms of various metabolic diseases, including diabetes. In a community study of 335 middle-aged women from different ethnicities, a cross-sectional analysis showed that more remarkable variability in bedtime was associated with increased insulin resistance (33). Irregular sleep is also linked to poor blood sugar control in individuals with diabetes. Studies in patients with type 1 diabetes (T1D) suggest that higher sleep duration and midpoint time variability are associated with poorer glycemic control after adjusting for covariates such as neurological symptoms, risk of sleep apnea, and self-reported poor sleep quality (34). In patients with type 2 diabetes (T2D), higher variability in self-reported and actigraphy-measured sleep duration is associated with higher HbA1c values (35, 36).

The link between the Sleep Regularity Index (SRI) and diabetes was also explored in the study. An analysis of data from the MESA study of 1978 older adults found that lower SRI values were associated with higher HbA1c and fasting glucose levels (16). In a US Hispanic/Latino study, cross-sectional results showed that lower SRI values were associated with an increased prevalence of diabetes, and the association was most pronounced in older adults. However, in the prospective analysis of this study, no significant associations of SRI values with glucose biomarkers and incidence of diabetes were observed (37).

Several studies have been carried out on the correlation between interdaily stability (IS) and diabetes. A study of 1,137 old adults demonstrated that subjects with higher IS values had a lower prevalence of diabetes (17). However, in participants without diabetes, IS was not associated with the level of HbA1c (17). Another population-based study 2,156 adults demonstrated that IS was not associated with HbA1c, insulin resistance, diabetes (22).

Social jetlag (SJL) is a sign of a mismatch between social schedules and biological clocks. The link between SJL and the development of diabetes has also been reported in multiple studies. One study noted that patients with diabetes had higher self-reported SJL than healthy individuals (35). Greater SJL was also associated with higher fasting glucose levels and insulin resistance among healthy middle-aged adults working full-time day shifts (38). Similar associations have been reported in other epidemiological studies (39–41). But in a cross-sectional study of 1,014 non-shift working adults with prediabetes, social jetlag was not associated with HbA1c levels (42). The association between social jet lag and blood sugar control in people with diabetes has also been studied. In two small samples of people with type 1 diabetes, social jet lag was associated with higher HBA1c levels (43, 44). The association between social jet lag and HBA1c was also found in a cross-sectional study of 225 patients with type 2 diabetes (45). It is important to note that age modifies the association between SJL and diabetes. In a cross-sectional analysis of data of 1,585 participants (mean age 60.8 years) from the New Hoorn Study cohort, the age-stratified analysis showed mixed results. Greater SJL was associated with a higher prevalence of diabetes in the subgroup younger than 61 but not in the subgroup older than 61 (46). Two related adolescent studies did not find an association between SJL and markers of glucose dysregulation (27, 28). In a cross-sectional study of 76 college students with T1D, social jetlag was not a significant predictor of HbA1c (47).

Overall, the association between SD of sleep duration or timing and glucose metabolism may differ across the population. SD of sleep characteristics or IS may be more consistently associated with HbA1c in patients with diabetes compared with the general population. Current evidence showed that SRI was significantly associated with diabetes, glucose and HbA1c.The link between SJL and glucose metabolism showed a clear age distribution. Possible explanations are given for the inverse U-shaped association between age and effect results. The effect of SJL on glucose metabolism may have a time-cumulative effect, and the more minor associations in adolescents may be attributable to less received exposure. Older adults, primarily in retirement, generally experience reduced SJL compared with younger adults. Furthermore, the strength of the association is attenuated due to poorer glucose regulation and a higher prevalence of chronic disease ascribed to physiological aging in older adults.

### 2.3. Obesity

Obesity is one of the most critical risk factors for cardiovascular disease and diabetes, affecting more than 600 million people worldwide. The etiology of obesity is complex, and the association of sleep regularity as a behavioral factor with obesity has been explored in several studies (Supplementary Table S3). Various methods have been established to assess obesity and its extent, including the most commonly used BMI and waist circumference (WC), bioelectrical impedance analysis, and imaging-based methods. The results of studies investigating associations between the standard deviation (SD) of sleep parameters and BMI were mixed. A large-scale retrospective cohort study of 21,148 participants showed that the variability of sleep duration is positively related to BMI (48). Similarly, greater variability in habitual sleep duration was associated with increased BMI in a cross-sectional study of 471 individuals (49). Two studies on elders also indicated that SD of actigraphy-derived sleep duration was associated with BMI (50, 51). The link between irregular sleep and BMI also exists in teens. One cross-sectional study recruiting 78 college students demonstrated that bedtime variability was related to BMI (52). Another study of 307 college students showed that greater variability in wake time was associated with higher BMI (53). In a study of children who were already obese, multivariate models showed that SD of sleep duration was significantly positively associated with both BMI and WC (54). Instead of these results, several studies did not observe a link between sleep and changes in BMI (30, 33, 55, 56). In clinical studies, obesity is usually defined as BMI ≥ 30 kg/m^2^. A cross-sectional study of 6,038 elderly adults (3,053 men and 2,985 women) was conducted to analyze the association between sleep regularity and obesity. The results showed that each hour of standard deviation in nighttime sleep duration increased the odds of obesity by 63% in men (OR = 1.63, 95% CI [1.31–2.02]) and by 22% in women (OR = 1.22, 95% CI) [1.01–1.47]) (57). In the cross-sectional analysis of actigraphy data from 2,598 subjects, higher sleep duration variability was more likely to be obesity (19). However, no significant association between sleep duration variability measured by actigraphy and obesity was found in 1986 community-dwelling elders (30). Several studies also examined the association between SD of sleep dimensions and WC, but no significant association was found (30, 51, 55).

Recent evidence maybe has shown a relationship between SRI and obesity. In the analysis of 1978 older adults from the Multi-Ethnic Study of Atherosclerosis (MESA) study, lower SRI was associated with higher BMI (16).

The literature on the association between interdaily stability (IS) and obesity is less consistent. In the cross-sectional analysis of 1,137 individuals, higher IS predicts increased rates of having obesity (17). However, no significant association between IS and BMI was observed (22).

The association between social jetlag (SJL) and obesity has also received extensive attention. A population-based European survey showed that SJL is associated with increased BMI (58). The findings are consistent with several subsequent studies (38, 40). Specifically, a cross-sectional analysis of data from 815 participants at age 38 showed that SJL was positively associated with BMI (40). Another study in 447 middle-aged adults (mean age 42.7 years) showed that SJL was associated with BMI (38). A study in the general population indicated that people with social jetlag>2 h had higher BMI compared with social jetlag<1 h (59). In comparison, multiple studies have found no association between social jet lag and BMI (24, 25, 43, 60). Interestingly, the association between social jet lag and BMI may be influenced by diurnal preference. Only in the participants with morning type, social jetlag was positively associated with BMI (61). Two studies showed correlation between obesity defined as BMI ≥ 30 kg/m^2^ and SJL (40, 61). However, no significant association was found between obesity and SJL in 4837 US adults (62). Current research also suggested that individuals with greater social jet lag are more likely to have larger WC (40, 41, 61). It is worth noting that the participants of the above studies were mainly adults. Regarding adolescents, multiple studies have not reported a clear association between SJL and measures of obesity (26, 28, 63). In a cross-sectional study of Latino minors, SJL was associated with healthier behaviors and lower odds of being overweight (28). The association between SJL and obesity also had gender differences. A study in adolescents showed that SJL was associated with higher levels of obesity only in girls (27).

In summary, SRI was significantly associated with BMI. Nevertheless, current evidence is inconsistent for the association between obesity and other metrics of sleep regularity. SD of sleep duration may be more likely related to BMI in comparison to sleep timing. The results on the association of SJL with obesity are mixed, with a strong and consistent association between SJL and obesity appearing in adults. In contrast, no significant association was observed between children and adolescents. Further large-scale prospective studies are needed to confirm the confounding effect of age.

### 2.4. Coronary heart disease

There has been less previous evidence for the link between irregular sleep and coronary heart disease (Supplementary Table S4). A study recruited 1992 participants without cardiovascular disease at baseline and conducted a median follow-up of 4.9 years. After adjusting for multiple cardiovascular risk factors, the hazard ratio for cardiovascular events was 2.14 (95% CI [1.24–3.68]) for individuals with sleep duration SD >120 min compared with individuals with sleep duration SD ≤60 min. Similarly, individuals with sleep onset SD >90 min were more likely to develop cardiovascular diseases than sleep onset SD ≤30 min (HR = 2.11, 95% CI [1.13–3.91]) (64). It should be noted that although the study did not explicitly analyze the association between coronary heart disease and irregular sleep, the Supplementary material showed that the cardiovascular events that occurred during the follow-up mainly consisted of coronary heart disease. A study among 1978 older adults demonstrated that greater sleep irregularity measured by SRI was correlated with 10-year risk of cardiovascular disease (16).

## 3. Mechanisms

**Figure 1:**
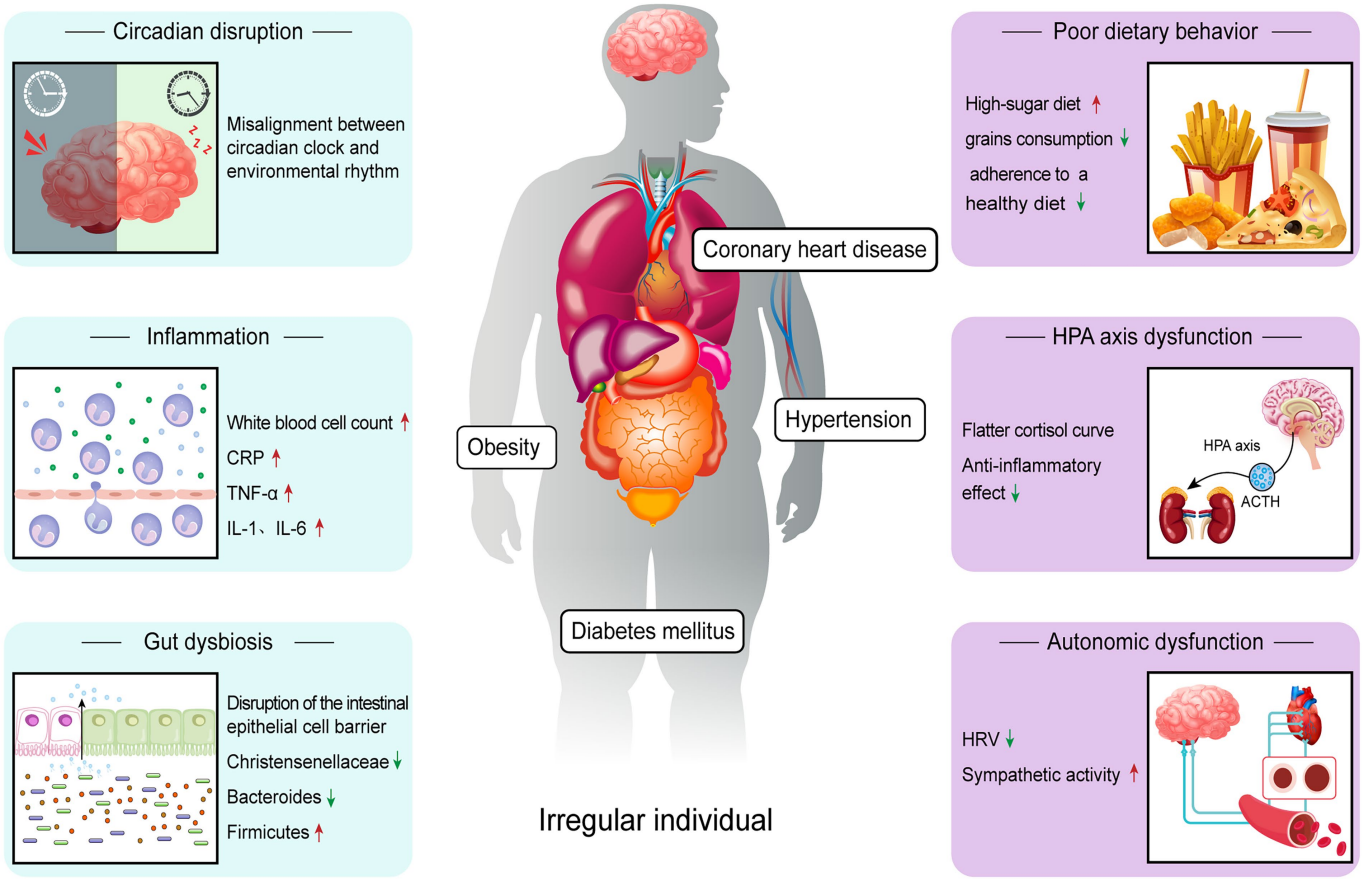
Relevant mechanisms through which irregular sleep increases risk of cardiometabolic disease. Irregular sleep induces changes in a variety of pathophysiological processes, including circadian disruption, autonomic dysfunction, inflammation, poor dietary behavior, HPA axis dysfunction and gut dysbiosis. Among them, circadian rhythm disturbance is considered to be the central link.

Individuals with irregular sleep show multiple pathophysiological changes (Figure 1), which may provide possible explanations for their increased risk of cardiometabolic disease.

### 3.1. Circadian dysfunction

Circadian dysfunction is thought to be the primary mechanism by which irregular sleep increases cardiometabolic risk. The circadian rhythm is the endogenous mechanism that coordinates physiological processes with biological behavior to synchronize with daily frequent environmental changes (65, 66). At the molecular level, it is manifested as the periodic expression of clock genes throughout the body (67, 68), about 24 h a cycle. It was found that almost all cardiovascular physiological parameters (69, 70) (including blood pressure, heart rate, endothelial function, and others) and metabolic parameters (71) were under the control of circadian rhythms and fluctuated regularly throughout the day, which is necessary to maintain normal body function. A growing body of evidence from animal models (72–74) and experimental human studies (75, 76) suggests that circadian rhythm impairment negatively affects cardiovascular function.

Circadian rhythms are regulated by external factors, such as light/dark alternations and feeding/fasting cycles. Recurring changes in a person’s sleep–wake schedule, along with irregular light exposure and eating timing, cause a misalignment between the internal circadian clock and the external exposure environment. The human body responds through a complex regulatory network, maintaining a dynamic balance between circadian and extrinsic rhythms (67). Nevertheless, when changes in sleep behavior are significant or persistent, they can exceed the superior limits of adjustment ability in body and lead to circadian dysfunction eventually.

Shift work exposes some workers to irregular sleep patterns. Studies showed higher levels of epigenetic methylation modifications (77–79) in clock genes and declined rhythm of melatonin and cortisol (80), suggesting that irregular sleep was associated with circadian dysfunction. Under the influence of the modern social lifestyle, people engaged in non-shift work also have a common phenomenon of irregular sleep. Although the degree of irregular sleep schedule is milder than shift workers, the long-term irregular sleep state may lead to chronic circadian dysfunction. A study of college students showed that irregular sleep and light patterns were associated with delayed circadian rhythms (6).

Irregular sleep causes circadian rhythms to disrupt, further exacerbating sleep–wake disorders. Damage to circadian rhythms is also associated with many pathophysiological processes, including autonomic nerve dysfunction (75, 81), increased inflammation (82), and metabolic disorders (83), all of which increase the risk of cardiovascular events. Therefore, circadian rhythm disturbance may be an essential and initial linkage in developing cardiometabolic diseases caused by irregular sleep.

### 3.2. Autonomic dysfunction

Autonomic dysfunction is another potential mechanism by which irregular sleep increases the risk of cardiometabolic disease. The autonomic nervous system is involved in physiological processes such as regulation of blood pressure (84), endothelial function (85), blood glucose, and lipid metabolism (86, 87). Autonomic dysfunction is associated with the progression of atherosclerosis. Heart rate variability (HRV) is a non-invasive measure widely used to detect autonomic function, and studies have shown that low HRV is associated with higher incidence and prevalence of coronary heart disease, hypertension (88, 89), diabetes (90), obesity (91, 92) in individuals.

A study in 421 healthy adolescents using actigraphy to measure sleep duration over multiple nights found that individuals with more significant variability in sleep duration exhibited lower HRV, suggesting a worse autonomic function. This association remained meaningful even after adjusting for sleep duration and efficiency (93). Furthermore, the findings showed that high sleep duration variability was more strongly associated with lower HRV than mean sleep duration (3, 94). However, further validation in other studies is lacking.

In addition, people with higher SJL showed lower HRV values during sleep (95). Of note, there was no significant difference in the expression of circadian markers between the two groups with high SJL and low, suggesting that high SJL can induce changes in autonomic function through other means. In animal model studies, experimental conditions showed that higher sympathetic activity in rats interfered with disturbed sleep patterns, leading to a higher degree of cardiac remodeling (96).

### 3.3. Inflammation

Inflammation is an integral part of the complex mechanisms involved in the occurrence and development of atherosclerosis. People with irregular sleep risk cardiometabolic disease, and inflammation may play an intermediate role. Higher levels of inflammatory markers represent a stronger inflammatory state. In one study, 42 healthy young adults were monitored for 14 days of activity recording, and the sleep regularity was described by the standard deviation of sleep duration and onset timing. Furthermore, it was found that irregular sleep was associated with significantly increased white blood cell count (97). Also, in another study, nocturnal variability in sleep duration was associated with higher levels of C-reactive protein (98), and similar results were found in a subsequent study with a larger sample (99). In a cohort of Mexico City adolescents, greater sleep duration variability was correlated with higher interleukin-1β (100). In addition to young adults or adolescents, a study in an elderly population showed that more significant variability in bedtime, later wake-up time, and more prolonged bedtime were all associated with higher tumor necrosis factor-α (101). However, a study of nurses only found that increased sleep duration variability was associated with higher levels of interleukin-1β and interleukin-6, not C-reactive protein and tumor necrosis factor-α (102). In a population with a large proportion of individuals diagnosed with obstructive sleep apnea syndrome (OSA), greater SJL was related to elevated levels of interleukin-1, after adjusting for OSA severity (103).

### 3.4. HPA axis dysfunction

Cortisol is a hormone the body produces in response to stress, and the HPA axis regulates its secretion. Under normal circumstances, cortisol secretion gradually declines after peaking in the morning. Normal cortisol rhythm plays a vital role in maintaining human health. Long-term irregular sleep patterns may be a constant stressor on the body, affecting the normal cortisol rhythm. Multiple studies have shown a link between sleep variability and poor cortisol rhythms. In a study of 76 adolescents, greater variability in sleep duration was associated with lower morning cortisol levels and a flatter cortisol curve (104). Similar results were confirmed in a larger sample (105).

Decreased cortisol hormone during wakefulness is not conducive to rapid recovery from sleep, which is associated with an increased incidence of mood disorders. Studies have shown that a flatter cortisol slope is associated with higher levels of coronary artery calcification (5) and increased cardiovascular mortality in nonclinical populations (106). Decreasing circadian cortisol slopes are also associated with increased future cardiac events and mortality in patients after coronary artery bypass grafting (107). The disruption of the circadian rhythm of cortisol weakens the anti-inflammatory effect, causing an overreaction of inflammation (108) and promoting the occurrence of cardiometabolic diseases.

### 3.5. Poor dietary habits

Diet provides the body with the energy and nutrients it needs. Poor dietary habits are associated with excess energy and unsuitable dietary structure, which increase the risk of cardiometabolic diseases (109, 110). Several studies have shown that irregular sleep increases total calorie intake. An adolescent study showed that higher sleep duration variability (HSV) was associated with poorer dietary habits, with an increase of 170 kcal in total daily energy intake for every 1-h increase in HSV (111). Studies on preschool children also showed similar results (112). In addition, multiple studies have linked irregular sleep patterns to undesirable dietary intake. In a study of 82 undergraduate students, more considerable objective SJL was associated with lower consumption of grains and greater consumption of sugar and confectioneries (113). Similarly, adolescents with larger SJL were linked with a higher frequency of sugary beverage consumption than those without SJL (114). Dietary patterns assess diets from a more holistic perspective. An epidemiological study showed that increased SJL was associated with lower healthy dietary pattern scores (115). A social survey conducted among Japanese workers revealed a negative correlation between SJL and adherence to a healthy diet (116). The Mediterranean diet is a dietary pattern related to better cardiovascular and metabolic health. A cross-sectional study of 534 young adults demonstrated that individuals with greater SJL showed lower adherence to the Mediterranean diet (117). In addition, irregular sleep can interfere with normal eating rhythms. The impaired eating rhythm and irregular sleep together cause the disturbance of the circadian rhythm, triggering a series of subsequent reactions and promoting the occurrence of cardiovascular and metabolic diseases.

### 3.6. Gut dysbiosis

Gut microbial imbalance sheds new light on the link between sleep regularity and cardiometabolic risk. The bacterial components of the gut microbiota and various secreted metabolites can be presented to human cells as signaling molecules to stimulate downstream metabolism-related pathways to participate in the metabolic regulation process (118, 119). Studies have demonstrated that gut microbial composition and function exhibit rhythmic fluctuations throughout the day (120, 121). This rhythmic change is compatible with intestinal mucosal epithelial cell biorhythms and feeding/fasting cycles, which promote metabolic health. Frequent changes in sleep patterns can cause disturbances in biological rhythms, often accompanied by disruption of eating rhythms and preferences for high-fat diets, which can interfere with a dynamically stable gut microbiota structure and established rhythm (122–124), resulting in adverse effects on the body. In rat experiments, circadian rhythm disturbances simulated by an 8-h circadian shift every 3 days can lead to imbalances in gut microbiota composition and rhythms (121), with reductions in the Christensenellaceae family attenuating such. The role of microbiota in suppressing body weight gain following a high-fat diet in the host (125). Increased numbers of Firmicutes and decreased numbers of Bacteroides were also observed in individuals who experienced irregular sleep. Increased Firmicutes to Bacteroides ratios are associated with weight gain and obesity (126). In addition, there is evidence that circadian rhythm dysregulation and sleep fragmentation can cause disruption of tight junctions in intestinal epithelial cells, leading to increased intestinal barrier permeability (127). Lipopolysaccharide (LPS) and other pro-inflammatory substances infiltrate the circulation, leading to systemic inflammation (128), thereby increasing the risk of obesity and insulin resistance.

## 4. Discussion

The link between sleep and cardiometabolic disease has received extensive attention. The number of studies on sleep regularity is limited compared to dimensions such as sleep duration and quality. In the modern lifestyle, situations such as shift work, sleep disorders, and electronic devices have made irregular sleep a widespread phenomenon, which needs more attention. Existing studies have mostly shown that irregular sleep increases the risk of cardiometabolic disease. However, the associations we observed were overwhelmingly cross-sectional, with few longitudinal studies to clarify causation. Based on the current status of this study, prospective study designs should be used in the future to explore the association between sleep regularity and cardiometabolic diseases.

Some studies did not draw positive conclusions, and the heterogeneity of sleep regularity evaluation indicators is one of the main reasons. A unified and more complete measurement to assess sleep regularity needs to be established in future research, and the newly emerged SRI is expected to become this representative indicator. In addition, some studies have shown age and gender differences in the correlation between irregular sleep and cardiometabolic disease. Future studies need to be conducted in a prospective and large-scale sample study to help formulate specific public health policies for different populations.

OSA is a common sleep disorder closely related to cardiovascular health. Patients with OSA are prone to sleep fragmentation and daytime sleepiness due to frequent apnea events at night. These symptoms make the sleep process of OSA patients lose a stable state and rhythm, leading to irregular sleep. OSA is an important confounding factor in exploring the relationship between sleep regularity and health. In future studies, we need to exclude patients diagnosed OSA when selecting study subjects or use statistical methods to balance the influence of OSA in multivariate regression analysis.

Notably, results of most existing studies on the link between sleep regularity and cardiovascular and metabolic diseases drew qualitative conclusions. Investigating the dosing-response relationship of irregular sleep on cardiometabolic health might prove important in future work. This will provide a theoretical basis for guiding the public to scientific sleep. Finally, how to improve irregular sleep is an important issue that we urgently need to solve. Sleep is an individual’s behavior primarily affected by subjective cognition and attitude. Future research is needed to clarify modifiable factors that affect sleep regularity. A study has improved sleep regularity scores of college students through strengthening education and information feedback to change their attitude towards sleep (129). In the future, we will need to identify other potential targets for improving irregular sleep.

In conclusion, irregular sleep can increase the risk of various cardiometabolic diseases, and multiple potential mechanisms explain this association. Sleep regularity is an essential dimension of sleep health that cannot be ignored. With the widespread prevalence of cardiometabolic diseases today, devoting much attention to the overall health of sleep may be a vital means to curb the epidemic trend. Clinicians and patients should be more attentive to the role of regular sleep on cardiometabolic health.

## Author contributions

Conception of the work from CZ and GQ. Article draft created from CZ. Critical revision of the article by GQ. All authors contributed to the article and approved the submitted version.

## Funding

This article was supported by the Shanxi Patent Transformation Project (No. 202201020) and Shanxi Special Project for Guiding the Transformation of Scientific and Technological Achievements (No. 201804D131045).

## Conflict of interest

The authors declare that the research was conducted in the absence of any commercial or financial relationships that could be construed as a potential conflict of interest.

## Publisher’s note

All claims expressed in this article are solely those of the authors and do not necessarily represent those of their affiliated organizations, or those of the publisher, the editors and the reviewers. Any product that may be evaluated in this article, or claim that may be made by its manufacturer, is not guaranteed or endorsed by the publisher.
