# Supplementary material for: Irregular sleep and cardiometabolic risk: Clinical evidence and mechanisms
Source: Front Cardiovasc Med. 2023 Feb 17;10:1059257. doi: 10.3389/fcvm.2023.1059257 (PMC9981680; doi:10.3389/fcvm.2023.1059257)
Supplement: Supplementary file 1 [file Table_1.DOCX]

Supplementary Table S1. Summary of studies examining the association between sleep regularity and hypertension

| Author (year) | Study Design | Participant Characteristics | Sleep Regularity Measure | Conclusion |
| --- | --- | --- | --- | --- |
| **Standard deviation（SD）** | | | | |
| Hausler, 2020  (19) | Cross-sectional study | 2598 subjects (mean age 61.9 years, 46.3% men) for cross-sectional analysis | Standard deviation of sleep duration (measured by actigraphy over 14 days) | No association was found between sleep duration variability and hypertension. |
| Huang, 2019  (20) | Cross-sectional and prospective study | 2003 subjects (mean age 69.5 years, 46.3% men) for cross-sectional analysis  970 subjects (mean age 66.7 years, 46.3% men) for prospective analysis | Standard deviation of sleep duration or sleep timing (measured by wrist actigraphy for 7 consecutive days) | No significant association was observed between sleep regularity measures and prevalence and incidence of hypertension. |
| Brindle, 2019  (21) | Cross-sectional study | 700 individuals | Standard deviation of actigraphy derived sleep midpoint | Sleep regularity was related to hypertension. |
| **Sleep Regularity Index (SRI)** | | | | |
| Lunsford-Avery, 2018  (16) | Cross-sectional study | 1976 US men and women from MESA study (mean age 68.7±9.2 years, 46% men) | SRI (measured by wrist actigraphy for 7 consecutive days) | Lower SRI was associated high blood pressure. |
| **Interdaily Stability Index (ISI)** | | | | |
| Abbott, 2019  (22) | Cross-sectional study | 1694 adults aged 18 to 64 years recruited from the Sueño ancillary study | Interdaily stability index (measured by wrist actigraphy for 7 days) | Higher interdaily stability was associated with increased prevalence of hypertension and higher blood pressure. |
| Sohaill, 2016  (17) | Cross-sectional study | 1137 individuals from the Rush Memory and Aging Project (mean age 81.6±7.5 years, 24%men) | Interdaily stability (measured by wrist actigraphy for at least 7 days) | Higher interdaily stability was associated with increased rates of having hypertension. |
| **Social jet lag (SJL)** | | | | |
| Rutters, 2014  (25) | Cross-sectional study | 145 healthy participants aged 18 to 55 years (67 men and 78 women) | Social jetlag (measured by Munich Chronotype Questionnaire) | No significant differences in blood pressure between groups was observed. |
| McMahon, 2018  (26) | Prospective study | 390 healthy adults (mean age 27.6±3.8 years, 49%men) | Social jetlag (measured by armband sensor for 6–10 days ) | No association was found between social jetlag and blood pressure. |
| Feliciano 2019  (27) | Cross-sectional study | 804 adolescents (mean age 13.2±0.9 years, 48%men) | Social jetlag (measured by wrist actigraphy for 7 to 10 consecutive days) | No association was observed between blood pressure and social jetlag. |
| Johnson, 2020  (28) | Cross-sectional study | 1208 Latino youth (mean age 12.3±0.4 years, 51%boys) | Social jetlag(self-reported) | No association was observed between social jetlag and odds of elevated blood pressure. |
| Mota, 2021  (23) | Retrospective study | 625 individuals with non-communicable chronic diseases (mean age 56.0 +12.0 years; 24% male) | Social jetlag(self-reported) | No linear association between social jet lag and blood pressure was found. |
| Mokhlesi, 2019  (24) | Cross-sectional study | 962 overweight/obese adults (mean age 52.2±9.5 years, 55% men) | Social jetlag(self-reported) | Social jetlag were independently associated with higher BP. |
