# Supplementary material for: Irregular sleep and cardiometabolic risk: Clinical evidence and mechanisms
Source: Front Cardiovasc Med. 2023 Feb 17;10:1059257. doi: 10.3389/fcvm.2023.1059257 (PMC9981680; doi:10.3389/fcvm.2023.1059257)
Supplement: Supplementary file 2 [file Table_2.DOCX]

Supplementary Table S2. Summary of studies examining the association between sleep regularity and diabetes

| Author (year) | Study Design | | Participant Characteristics | | Sleep Regularity Measure | | Conclusion | |
| --- | --- | --- | --- | --- | --- | --- | --- | --- |
| **Standard deviation（SD）** | | | | | | | | |
| Rosique-Esteban, 2018  (30) | Cross-sectional study | | 1986 community-dwelling elders (mean age 65.0±4.9 years, 53.0% men) | | Standard deviation of sleep duration (measured by 8 consecutive 24-hour days) | | Sleep variability was associated with diabetes, not FPG and HbA1c. | |
| Slavish, 2019  (31) | Cross-sectional study | | 771 adults (mean age 53.8 years, 49.3% men) | | Standard deviation of total sleep duration (measured by wrist actigraphy for 14 days) | | No significant association was observed between sleep regularity and diabetes. | |
| Hausler, 2020  (19) | Cross-sectional study | | 2598 subjects (mean age 61.9 years, 46.3% men) for cross-sectional analysis | | Standard deviation of sleep duration (measured by actigraphy over 14 days) | | No association was found between sleep duration variability and diabetes. | |
| Huang, 2019  (20) | Cross-sectional and prospective study | | 2003 subjects (mean age 69.5 years, 46.3% men) for cross-sectional analysis  970 subjects (mean age 66.7 years, 46.3% men) for prospective analysis | | Standard deviation of sleep duration or sleep timing (measured by wrist actigraphy for 7 consecutive days) | | Cross-sectionally, greater sleep regularity was associated with higher fasting glucose; prospectively, no significant association was found between sleep regularity and higher fasting glucose. | |
| Taylor, 2016  (33) | Cross-sectional study and prospective study | | 335 participants from the SWAN Sleep Study (mean age 52.1±2.1 years, 100%women) | | Standard deviation of bedtime (measured by sleep diary for 11-14nights) | | Cross-sectionally, greater variability in bedtime was associated with higher HOMA-IR; prospectively, greater bedtime delay predicted increased HOMA-IR at follow-up time. | |
| Soltero, 2022  (32) | Cross-sectional study | | 38 adolescents (aged 12-16 years) and 22 young adults (aged 18-22 years) | | Standard deviation of sleep duration (measured by wrist actigraphy for 7 days) | | Sleep regularity was negatively associated with fasting and 2-hour glucose in young adults. | |
| Chontong, 2016  (34) | Cross-sectional study | | 41 adult patients with type 1 diabetes (mean age 52.1±14.8 years, 39% men) | | Standard deviation of sleep duration and mid-sleep time (measured by wrist actigraphy for 5 days) | | Higher sleep variability was significantly associated with poorer glycemic control. | |
| Zhu, 2020  (36) | Cross sectional study | | 56 adults with type 2 diabetes (mean age 60.7 years, 44.6% men) | | Standard deviation of sleep duration (measured using the Consensus Sleep Diary over 8 days) | | Greater sleep regularity predicts higher hemoglobin A1C. | |
| Kelly, 2022  (35) | Cross sectional study | | 27 healthy controls and 30 adults with  well-controlled uncomplicated type 2 diabetes mellitus | | Standard deviation of sleep timing (measured by wrist actigraphy for an average of 9 days) | | Variability on sleep duration is associated with higher HbA1c values | |
| **Interdaily Stability Index (ISI)** | | | | | | | | |
| Sohaill, 2016  (17) | | Cross-sectional study | | 1137 individuals from the Rush Memory and Aging Project (mean age 81.6±7.5 years, 24%men) | | Interdaily stability (measured by wrist actigraphy for at least 7 days) | | Higher interdaily stability was associated with increased rates of having diabetes. |
| Abbott, 2019  (22) | | Cross-sectional study | | 1694 adults aged 18 to 64 years recruited from the Sueño ancillary study | | Interdaily stability index (measured by wrist actigraphy for 7 days) | | Interdaily stability index was not associated with log of homeostatic assessment of insulin resistance, or log of glycosylated hemoglobin. |
|  | |  | |  | |  | |  |
| **Sleep Regularity Index (SRI)** | | | | | | | | |
| Lunsford-Avery, 2018  (16) | Cross sectional study | | 1976 US men and women from MESA study (mean age 68.7±9.2 years, 46% men) | | SRI (measured by wrist actigraphy for 7 consecutive days) | | Lower SRI was associated with higher hemoglobin A1C, and fasting blood glucose. | |
| Fritz, 2021  (37) | Cross-sectional study and prospective study | | 2107 adults from the Sueño ancillary study of HCHS/SOL (mean age 40.7±0.4 years, 48.9%men) | | SRI (measured by wrist actigraphy for 7 days) | | Cross-sectionally, lower SRI was associated with higher odds of diabetes; prospectively, no statistically significant associations were found between SRI and diabetes incidence, as well as baseline HOMA-IR, HOMA-β, and HbA1c values. | |
| **Social jetlag (SJL)** | | | | | | | | |
| Aguayo, 2022  (39) | Cross-sectional study | | 1028 participants (mean age 51.7±12.2 years, 45.3% men) | | Social jetlag (measured by wrist actigraphy for 7days) | | Increased social jetlag were associated with higher HbA1c, not associated with insulin sensitivity. | |
| Larcher, 2016  (44) | Cross-sectional study | | 80 adult patients with type 1 diabetes (46% female) | | Social jetlag (measured by wrist actigraphy for 7days) | | Social jetlag was associated with HbA1c. | |
| Saylor, 2019  (47) | Cross-sectional study | | 76 college students with type 1 diabetes (mean age 20.4±1.6 years, 75.7% men) | | Social jetlag(self-reported) | | Social jetlag was not a significant predictor of HbA1c. | |
| Islam, 2018  (41) | Cross-sectional study | | 1164 Japanese employees aged 18-78 years | | Social jetlag(self-reported) | | Greater social jetlag was significantly associated with an increased likelihood of having higher fasting blood glucose. | |
| Kelly, 2022  (35) | Cross sectional study | | 27 healthy controls and 30 adults with  well-controlled uncomplicated type 2 diabetes mellitus | | Social jetlag (measured by wrist actigraphy for an average of 9 days) | | Greater self-reported social jetlag in the diabetes group, but no association was observed between social jetlag and HOMA-IR. | |
| Wong, 2015  (38) | Cross-sectional study | | 447 healthy, midlife adults (mean age 42.7 years; 43% male) | | Social jetlag (measured by wrist actigraphy for 7 days) | | Greater SJL related to higher fasting plasma insulin and insulin resistance. | |
| Koopman, 2017  (46) | Cross-sectional study | | 1585 adults from the New Hoorn Study cohort (mean age 60.8±6 years, 47% men) | | Social jetlag (measured by the Munich Chrono type Questionnaire) | | No significant association between social jetlag status and diabetes/prediabetes. | |
| Feliciano, 2019  (27) | Cross-sectional study | | 1208 Latino youth (mean age 12.3±0.4 years, 51%boys) | | Social jetlag (measured by wrist actigraphy for 7 to 10 consecutive days) | | No significant association was observed between social jetlag and HOMA-IR. | |
| Johnson, 2020  (28) | Cross-sectional study | | 1208 Latino youth (mean age 12.3±0.4 years, 51%boys) | | Social jetlag(self-reported) | | No significant association was observed between social jetlag and measures of glucose. | |
| Kelly, 2020  (45) | Cross-sectional study | | 252 type 2 diabetes patients (mean age 61.9±10.5 years, 67% male) | | Social jetlag (measured by the Munich Chrono type Questionnaire) | | SJL was associated with glycemic control, independently of other predictors. | |
| Rusu, 2019  (43) | Cross-sectional study | | 115 type 1 diabetes patients | | Social jetlag (self-reported) | | SJL is associated with poor glycemic control independent of sleep quality, sleep duration, and chronotype. | |
| Parsons, 2015  (40) | Cross-sectional study | | 815 non-shift workers | | Social jetlag (measured by the Munich Chrono type Questionnaire) | | Social jetlag was associated with HbA1c controlling for sex、chronotype and sleep duration. | |
| Anothaisintawee, 2017  (42) | Cross-sectional study | | 1014 non-shift working adults with prediabetes | | Social jetlag | | Social jetlag was not associated with HbA1c. | |
