# Supplementary material for: Irregular sleep and cardiometabolic risk: Clinical evidence and mechanisms
Source: Front Cardiovasc Med. 2023 Feb 17;10:1059257. doi: 10.3389/fcvm.2023.1059257 (PMC9981680; doi:10.3389/fcvm.2023.1059257)
Supplement: Supplementary file 3 [file Table_3.DOCX]

Supplementary Table S3. Summary of studies examining the association between sleep regularity and obesity

| Author (year) | Study Design | Participant Characteristics | Sleep Regularity Measure | Conclusion |
| --- | --- | --- | --- | --- |
| **Standard deviation（SD）** | | | | |
| Patel，2014  (57) | Cross-sectional study | 3053 men (mean age 76.4) participating in the Osteoporotic Fractures in Men Study and 2985 women (mean age 83.5) participating in the Study of Osteoporotic Fractures | Standard deviation of nocturnal sleep duration (measured by wrist actigraphy over 5 consecutive days) | Greater variability in sleep duration was associated with an increased likelihood of obesity and BMI. |
| Kobayashi, 2013  (48) | Retrospective cohort study | 21,148 participants (mean age 51±12years, 49.6% men) | Standard deviation of sleep duration (measured by questionnaire annually for 3 years) | The variability of sleep duration is positively related to BMI. |
| Ogilvie, 2016  (50) | Cross-sectional study | 2146 participants from the MESA Sleep study (mean age 68.6±9.2 years, 46% men) | Standard deviation of sleep duration (measured by wrist actigraphy for 7days) | Greater sleep variability was associated with higher BMIs, larger waists, and more body fat. |
| Häusler, 2020  (19) | Cross-sectional study | 2598 subjects (mean age 61.9 years, 46.3% men) for cross-sectional analysis | Standard deviation of sleep duration (measured by actigraphy over 14 days) | Individuals with higher sleep variability were more likely to be obese. |
| Nicholson, 2021  (53) | Cross-sectional study | 307 college students (mean age 18.9±0.9 years,15.3% men) | Standard deviation of sleep duration, bedtime, waketime (measured by online dairy for 7 days) | Greater variability in wake time was associated with higher BMI adjusting for gender, depressive symptoms, and average total sleep duration. |
| Kim, 2015  (49) | Cross-sectional study | 191 elderly women (mean age 83.4±2.6 years) | Standard deviation of sleep duration, bedtime, sleep midpoint (measured by actigraphy over 5 days) | The analysis revealed significant associations between bedtime variation and body composition. |
| Taylor, 2016  (33) | Cross-sectional study and prospective study | 335 participants from the SWAN Sleep Study (mean age 52.1±2.1 years, 100%women) | Standard deviation of bedtime (measured by sleep diary for 11-14nights) | After adjusting for covariates, bedtime variability was not associated with BMI. |
| Chan, 2017  (52) | Cross-sectional study | 78 college students | Standard deviation of sleep duration, wake time, bedtime (measured by actigraphy for 7 days) | Only bedtime variability was related to BMI. |
| Papandreou, 2020  (51) | Prospective study | 1986 community-dwelling elders (mean age 65.0±4.9 years, 53.0% men) | Standard deviation of sleep duration (measured by actigraphy for 8 consecutive 24-hour days) | Sleep variability was associated with BMI, not WC. |
| Schreiber, 2019  (55) | Cross-sectional study | 132 midlife women (mean age 52.9±6.9 years) | Standard deviation of sleep timing(measured by actigraphy) | Sleep variability was not associated with BMI or waist circumference. |
| Bowman, 2020  (56) | Cross-sectional study and prospective study | 221 midlife women | Standard deviation of sleep midpoint (measured by actigraphy) | Sleep variability was not associated with BMI or waist circumference in adjusting model. |
| Rosique-Esteban, 2018  (30) | Cross-sectional study | 1986 community-dwelling elders (mean age 65.0±4.9 years, 53.0% men) | Standard deviation of sleep duration (measured by 8 consecutive 24-hour days) | Sleep variability was not associated with BMI, WC and the prevalence of obesity. |
| Zhou, 2018  (54) | Cross sectional study | 188 children with obesity (mean age 10.5±1.4 years, 43%men) | Standard deviation of sleep duration (measured by wrist actigraphy for multiple days) | Greater variability in bedtime and wake time are associated with greater adiposity. |
| **Interdaily Stability Index (ISI)** | | | | |
| Sohaill, 2016  (17) | Cross-sectional study | 1137 individuals from the Rush Memory and Aging Project (mean age 81.6±7.5 years, 24%men) | Interdaily stability (measured by wrist actigraphy for at least 7 days) | Higher interdaily stability was associated with increased rates of having obesity. |
| Abbott, 2019  (22) | Cross-sectional study | 1694 adults aged 18 to 64 years recruited from the Sueño ancillary study | Interdaily stability index (measured by wrist actigraphy for 7 days) | The association between interdaily stability index and BMI was not significant. |
| **Sleep Regularity Index (SRI)** | | | | |
| Lunsford-Avery, 2018  (16) | Cross-sectional study | 1976 US men and women from MESA study (mean age 68.7±9.2 years, 46% men) | SRI (measured by wrist actigraphy for 7 consecutive days) | Lower SRI was associated greater obesity independent of sleep duration. |
| **Social jet lag (SJL)** | | | | |
| Roenneberg, 2012  (58) | Cross sectional study | Large-scale epidemiological study | Social jetlag (measured by the Munich Chronotype Questionnaire) | Greater social jetlag was associated with increased BMI. |
| Rutters, 2014  (25) | Cross-sectional study | 145 healthy participants aged 18 to 55 years (67 men and 78 women) | Social jetlag (measured by Munich Chronotype Questionnaire) | Social jetlag was not associated with BMI and WC. |
| Parsons, 2015  (40) | Cross sectional study | 815 non-shift workers | Social jetlag (measured by the Munich Chronotype Questionnaire) | Greater social jetlag scores had higher average BMI and more fat mass. |
| Wong, 2015  (38) | Cross sectional study | 447 midlife adults (mean age 42.7 years, 47%men) | Social jetlag (measured by wrist actigraphy) | Greater social jetlag was related to a higher adiposity. |
| Suikki, 2021  (61) | Cross sectional study | 6779 Finns (45% men, aged 25-74 years) | Social jetlag | Only in the participants with morning type, social jetlag was associated with obesity and higher BMI, WC. |
| Anothaisintawee, 2018  (60) | Cross sectional study | 2,133 patients with prediabetes(mean age 63.6±9.2 years, 34.3%men) | Social jetlag(self-reported) | Social jetlag was not associated with BMI. |
| Islam, 2018  (41) | Cross-sectional study | 1164 Japanese employees aged 18-78 years | Social jetlag(self-reported) | Greater social jetlag was significantly associated with an increased likelihood of having high waist circumference. |
| Rusu, 2019  (43) | Cross-sectional study | 115 type 1 diabetes patients | Social jetlag (self-reported) | Social jetlag was not associated with BMI. |
| McMahon, 2019  (26) | Prospective study | 390 healthy adults (mean age 27.6±3.8 years, 49%men) | Social jetlag (measured by armband sensor for 6–10 days) | No association between social jetlag and obesity was found. |
| Mokhlesi, 2019  (24) | Cross-sectional study | 962 overweight/obese adults (mean age 52.2±9.5 years, 55% men) | Social jetlag(self-reported) | Social jetlag was not associated with BMI. |
| Hawkins, 2021  (62) | Cross sectional study | 4837 US adults | Social jetlag(self-reported) | No significant association was found between obesity and social jetlag. |
| Johnson, 2021  (28) | Cross-sectional study | 1208 Latino youth (mean age 12.3±0.4 years, 51%boys) | Social jetlag(self-reported) | Greater social jetlag was associated with lower odds of being overweight. |
| LeMay-Russell, 2021  (63) | Prospective study | 137 youths (mean age 12.5±2.6years, 46% men) | Social jetlag (measured by wrist actigraphy) | No association was observed between social jetlag with 1-year fat mass. |
| Feliciano, 2019  (27) | Cross-sectional study | 804 adolescents (mean age 13.2±0.9 years, 48%men) | Social jetlag (measured by wrist actigraphy for 7 to 10 consecutive days) | Greater social jetlag was associated with higher waist circumference ang higher fat mass. |
