# Supplementary material for: Irregular sleep and cardiometabolic risk: Clinical evidence and mechanisms
Source: Front Cardiovasc Med. 2023 Feb 17;10:1059257. doi: 10.3389/fcvm.2023.1059257 (PMC9981680; doi:10.3389/fcvm.2023.1059257)
Supplement: Supplementary file 4 [file Table_4.DOCX]

Table 2. Description of study examining the association between sleep regularity and CAD.

| Author (year) | Study Design | Participant Characteristics | Sleep Regularity Measure | Conclusion |
| --- | --- | --- | --- | --- |
| Huang, 2020  (64) | Prospective study | 1992 participants free of CVD | Standard deviation of sleep duration and sleep onset timing (measured by wrist actigraphy across 7days) | Greater variability in sleep duration and sleep onset was associated higher incidence of CAD. |
| Lunsford-Avery, 2018  (16) | Cross-sectional study | 1976 US men and women from MESA study (mean age 68.7±9.2 years, 46% men) | SRI (measured by wrist actigraph for 7 consecutive days) | Lower SRI was associated higher 10-year risk of cardiovascular disease. |
